# The added value of using convective-permitting regional climate model simulations to represent cloud band events over South America

**DOI:** 10.1007/s00382-024-07460-3

**Published:** 2024-10-16

**Authors:** Marcia T. Zilli, Murilo Ruv Lemes, Neil C. G. Hart, Kate Halladay, Ron Kahana, Gilberto Fisch, Andreas Prein, Kyoko Ikeda, Changhai Liu

**Affiliations:** 1 School of Geography and the Environment, University of Oxford, S Park Rd, Oxford, Oxfordshire OX1 3QY UK; 2 CPTEC, INPE, Av. dos Astronautas, 1758, São José dos Campos, SP 12227-010 Brazil; 3 Department of Geography, Manchester University, Manchester, 610101 UK; 4 Met Office, FitzRoy Rd, Exeter, Devon EX1 3PB UK; 5 Department of Agricultural Sciences, University of Taubaté, Estr. Mun. Prof. Dr. José Luís Cembraneli, 5000, Taubaté, SP 12081-010 Brazil; 6 U.S. National Science Foundation National Center for Atmospheric Research, 1850 Table Mesa Dr., Boulder, CO 80305 USA; 7 Institute for Atmospheric and Climate Science, ETH Zürich, Zurich, Switzerland

**Keywords:** Convective-permitting models, South America, SACZ, Rainfall rates, Model bias

## Abstract

Climate science has long explored whether higher resolution regional climate models (RCMs) provide improved simulation of regional climates over global climate models (GCMs). The advent of convective-permitting RCMs (CPRCMs), where sufficiently fine-scale grids allow explicitly resolving rather than parametrising convection, has created a clear distinction between RCM and GCM formulations. This study investigates the simulation of tropical-extratropical (TE) cloud bands in a suite of pan-South America convective-permitting Met Office Unified Model (UM) and Weather Research and Forecasting (WRF) climate simulations. All simulations produce annual cycles in TE cloud band frequency within 10–30% of observed climatology. However, too few cloud band days are simulated during the early summer (Nov–Dec) and too many during the core summer (Jan–Feb). Compared with their parent forcing, CPRCMs simulate more dry days but systematically higher daily rainfall rates, keeping the total rain biases low. During cloud band systems, the CPRCMs correctly reproduced the observed changes in tropical rain rates and their importance to climatology. Circulation analysis suggests that simulated lower subtropical rain rates during cloud bands systems, in contrast to the higher rates in the tropics, are associated with weaker northwesterly moisture flux from the Amazon towards southeast South America, more evident in the CPRCMs. Taken together, the results suggest that CPRCMs tend to be more effective at producing heavy daily rainfall rates than parametrised simulations for a given level of near-surface moist energy. The extent to which this improves or degrades biases present in the parent simulations is strongly region-dependent.

## Introduction

Convective-Permitting Regional Climate Models (CPRCMs) are regional climate models running at kilometre-scale horizontal grid-spacing ($\sim 4$ km or less). The higher resolution of those models enables explicitly resolving deep convective processes, even though small-scale processes are still under-resolved (Prein et al. 2015; Lucas-Picher et al. 2021; Halladay et al. 2023), and can improve the representation of processes related to topographic features and land-cover contrasts (Rowell and Berthou 2023). Previous studies demonstrated that CPRCM simulations improve the representation of the precipitation diurnal cycle and extremes (e.g. Prein et al. 2015; Stratton et al. 2018; Halladay et al. 2023), including a better representation of tropical-extratropical (TE) cloud bands over Southern Africa (Hart et al. 2018). Here, we will evaluate the added value of CPRCM simulations in representing TE cloud band events over South America (SAm).

TE cloud bands are the main source of precipitation during the rainy season (November–March) over SAm (Zilli and Hart 2021). When located over Eastern Brazil, these events can persist for 4 or more days and contribute to more than 60% of the rainy season precipitation despite being active in less than 30% of the days (Zilli and Hart 2021). The presence of persistent cloud band events, known regionally as South Atlantic Convergence Zone (SACZ) events, increases the risk of natural disasters (da Fonseca and Cataldi 2021) while their absence is related to droughts, such as the one observed in Eastern Brazil in 2013–2015 (Coelho et al. 2016a, b; Cünningham 2020). Cloud band events of shorter duration are more common during the onset (September to November) or demise (after March) of the rainy season and can contribute to $\sim$ 15% of the total precipitation, especially over subtropical SAm (Zilli and Hart 2021).

The presence of cloud bands is modulated by the interplay between tropical and extratropical forcings (Zilli and Hart 2021). Mid-latitude disturbances from the extratropics interact with the upper-level wind anomalies over the subtropics, modifying the location and persistence of the cloud bands. When upper-level westerlies prevail in the basic-state subtropical flow, the midlatitude disturbances propagate further into the tropics, resulting in more intense and persistent cloud band events (Zilli and Hart 2021), including the SACZ (Kodama 1992, 1993; Carvalho et al. 2011; Gonzalez and Vera 2014). Conversely, weaker westerlies or easterlies in upper-level winds over the subtropics shift the critical line for Rossby wave (RW) propagation further south, hindering any further propagation of the mid-latitude disturbances. In these cases, the cloud bands are located over subtropical latitudes and last up to three days (Zilli and Hart 2021). The convection during these events is modulated by the moisture transported from the Amazon by the Low-Level Jet (LLJ) east of the Andes. This circulation is typical of the inactive phase of the SACZ (Gonzalez and Vera 2014; Mattingly and Mote 2017).

Thus, to correctly simulate the location and persistence of cloud band events, climate models should be able to reproduce dynamic and thermodynamic aspects of the South American rainy season, including the propagation of mid-latitude disturbances, the convection over the Amazon, and the southward transport of moisture by the LLJ. Previous studies identified a gradual evolution in the representation of the South American rainy season climatology, including the SACZ and the LLJ, by global climate models participating in the successive phases of the Climate Model Intercomparison Project (CMIP; Gulizia and Camilloni 2015; Zilli and Carvalho 2021; Firpo et al. 2022). Nonetheless, these models have issues representing the location and intensity of the LLJ, resulting in biases in the precipitation intensity over central Brazil (Firpo et al. 2022) and subtropical SAm (Gulizia and Camilloni 2015; Almazroui et al. 2021).

Two models that reproduce the main characteristics of the seasonal precipitation and circulation over SAm, including the LLJ and the SACZ are the Brazilian Global Atmospheric Model version 1.2 (BAM-1.2) and the Hadley Centre Global Environment Model in the Global Coupled configuration 3.1 (HadGEM3-GC3.1) (Monerie et al. 2020; García-Franco et al. 2020; Coelho et al. 2021, 2022). Zilli et al. (2023) demonstrated that both these models reproduced the key dynamical aspects related to the development of cloud band events. Nonetheless, these models have biases in the precipitation during the cloud band events and simulate a weaker cloud band activity during the onset of the rainy season (September–December). These biases emerge from misrepresenting convective processes over the continent and the interaction between the large-scale circulation and the SAm topography due to the lower resolution of global climate models (Prein et al. 2015; Lucas-Picher et al. 2021). Approaches such as dynamic downscaling through regional climate models provide simulations with higher spatial resolution but still have limitations related to the sub-grid parametrisation (Prein et al. 2015) and uncertainties related to the lateral boundary conditions (Ambrizzi et al. 2019).

Currently, few regional-scale convective-permitting simulations are available over SAm, most of them related to the Coordinated Regional Climate Downscaling Experiments (CORDEX) Flagship Pilot Study in Southeastern South America (FPS-SESA; Bettolli et al. 2021). In general, these are weather-like simulations (i.e., covering a period of a few days) run over a subcontinental area nested in a regional climate model (Bettolli et al. 2021; Lavin-Gullon et al. 2021; Feijoó and Solman 2022). In these studies, the use of a regional climate model did not improve the simulation of an extremely wet season, but the convective-permitting simulations better captured the spatial distribution of the accumulated precipitation (Bettolli et al. 2021).

Halladay et al. (2023) documents the recently completed Met Office 10-year simulation of a continental-scale CPRCM over SAm, with improvements in the representation of the precipitation frequency, intensity, and diurnal cycle despite overestimating the frequency of intense precipitation. This simulation builds on a similar continental scale simulation over Africa, CP4-Africa (Stratton et al. 2018), which saw similar improvements in rainfall characteristics (Berthou et al. 2019). Notably, the CP4-Africa simulation better represents the mean state of the subtropics of southern Africa (relative to the driving simulation), leading to a halving of regional wet season rainfall bias and improved representation of the annual cycle of synoptic-scale TE cloud bands (Hart et al. 2018). A similar modelling effort led by the South America Affinity Group from the National Center for Atmospheric Research (SAAG-NCAR) produced a 20-year retrospective continental scale CPRCM simulation considering a similar domain (Rasmussen et al. 2022; Liu et al. 2022; Dominguez et al. 2023).

This paper explores the representation of tropical-extratropical cloud bands in these two first-of-their-kind CPRCM simulations covering SAm and proposes to answer the following scientific questions:

- What is the impact of explicit convection on the representation of rainfall during tropical-extratropical cloud bands?
- To what extent does the explicit representation of convection change the simulation of the cloud band annual cycle over SAm relative to their driving simulation?

Details of the model simulations and observational data used in addressing these questions are provided next. Simulated climatological biases discussed in Sect. 3 provide the baseline for the TE cloud band event-based results in Sect. 4. Linking of these event characteristics to larger-scale dynamic and thermodynamics features is detailed in Sect. 5. Section 6 provides a broader synthesis of the detailed results sections and summarises the findings of this study.

## Data sets and methodology

### Data sets

**Fig. 1:**
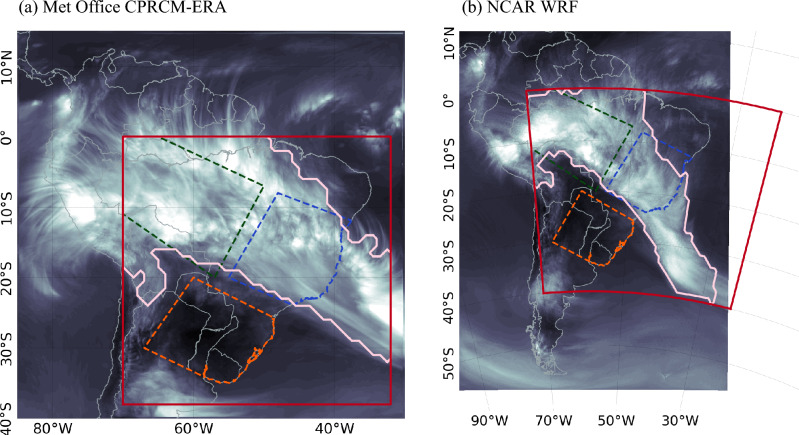
CPRCM domains and Region of Interest for cloud band identification (red squares). Daily mean OLR for 02-01-2000 in (**a**) UKMO CPRCM-ERA and (**b**) NCAR WRF, representing a day with an active SACZ (shading, low OLR values in whiter shades) and the cloud band signature as identified by the detection algorithm (light pink contour), with a threshold of (**a**) $240~W m^{-2}$ and (**b**) $250~W m^{-2}$. Dashed polygons represent the Southern Amazon (green), Eastern Brazil (blue) and Southeastern South America (orange)

Here, we examine CPRCM simulations produced by the UK Met Office (UKMO; MOHC-HadREM3-RAL1T-4.5 km) and NCAR. The UKMO Unified Model (UM) version 10.6 is used for two convective-permitting simulations. A hindcast (CPRCM-ERA) is nested within the domain of a UKMO regional climate model (MOHC-HadREM3-GA71-25 km, RCM hereafter) driven by ERA-Interim reanalysis (Dee et al. 2011). This nesting is required to support the step down from ERA-Interim horizontal resolution ($\sim 75~km)$ to the CPRCM grid spacing (4.5 *km*). The second simulation is for present-day climate (CPRCM-PD), directly nested in the UKMO atmosphere-only global climate model (MOHC-HadGEM3-GA7GL7-N512, GCM hereafter). Both CPRCMs (-ERA, -PD) cover the 10-year period of 1998–2007 with a horizontal grid spacing of 4.5 *km* over tropical and subtropical SAm ($85^{\circ }W; 40^{\circ }S$ to $30^{\circ }W; 15^{\circ }N$; Fig. 1a) with the convective parametrisation scheme disabled. The RCM and GCM have a horizontal grid spacing of 25 *km*, with convection parametrised by the Gregory and Rowntree (1990) scheme. Halladay et al. (2023) provide a detailed description of these model configurations. It is worth noting that the UKMO CPRCM configuration used here is very similar to that used for the 10-year convective-permitting climate simulations performed by Stratton et al. (2018) over Africa.

We also analysed the CPRCM simulations produced by the SAAG-NCAR (Liu et al. 2022; Rasmussen et al. 2022; Dominguez et al. 2023). This simulation uses the Weather Research and Forecasting (WRF) model version 4.1.5 forced by the European Centre for Medium-Range Weather Forecasts (ECMWF) fifth-generation reanalysis (ERA5; Hersbach et al. 2020). It covers 22 years (2000–2021) with a horizontal grid spacing of 4 *km* over the continental SAm and nearby waters (Fig. 1b). More details about this simulation can be found at https://ral.ucar.edu/projects/south-america-affinity-group-saag/model-output and Dominguez et al. (2023). Due to the different spatial coverage and simulation periods, we do not directly compare the simulations of these two CPRCMs. However, evaluating similarities and differences between these simulations, with distinct dynamical cores and lateral boundary conditions, can highlight simulation changes due to explicitly resolving the convection.

The characteristics of the simulated cloud bands are compared to those identified using observational data sets, considering the same period of each simulation (1998–2007 for the UKMO and 2000–2022 for NCAR WRF). To identify the observational cloud band events, we use the daily mean outgoing long-wave radiation (OLR) Version 1.2 data set provided by the National Oceanic and Atmospheric Administration (NOAA) Climate Data Record (CDR; Lee and Program 2011; Lee 2014). To characterise observed cloud band event precipitation, we considered three different sources: ERA5 (Hersbach et al. 2020); the Climate Hazards Center Infrared Precipitation with Station data (CHIRPS; Funk et al. 2015), a gridded rainfall product obtained by merging satellite imagery and in-situ station data; and the Brazilian Daily Weather Gridded Data (Br-DWGD), a gridded data set based on station-observed precipitation from Brazil (Xavier et al. 2022). Note that ERA5 is also the forcing data for the NCAR-WRF simulations. To avoid confusion, we will use the term “ERA5-NCAR” when indicating the forcing data for the NCAR-WRF. Otherwise, this data set will be indicated simply as “ERA5”. We will only show the precipitation results using CHIRPS, mainly because of its coverage over the entire continent, but will comment whenever the results using other precipitation data sets are relevant. Since Br-DWGD has data only over Brazil, we will not use this data set when analysing results over Southeastern South America.

The moist thermodynamic structure of the atmosphere is characterized by the moist static energy (MSE) and the vertically integrated water vapour transport (IVT). The MSE is estimated as:

1 $$\begin{aligned} MSE = c_pT+gz+L_vq \end{aligned}$$

where *T* is the temperature (in *K*), *z* is the geopotential height (in *m*), and *q* is the specific humidity (in $kg\,kg^{-1}$). $c_p$ is specific heat at constant pressure ($c_p = 1004\,J\,kg^{-1}\,K^{-1}$), $L_v$ is the latent heat of vaporisation ($L_v = 2500\,kJ.K^{-1}$), and *g* is the gravity ($g = 9.81\;m\,s^{-2}$). Equation 1 is calculated at 925 hPa, 850 hPa, 500 hPa, and 200 hPa.

The IVT is calculated as follows:

2 $$\begin{aligned} IVT = \left[ \left( \frac{1}{g} \int _{p_0}^{p_1} qu \,dp \right) ^2+\left( \frac{1}{g}\int _{p_0}^{p_1} qv \,dp \right) ^2 \right] ^{1/2} \end{aligned}$$

where *u* and *v* are the zonal and meridional components of the wind, respectively (in $m\,s^{-1}$). The transport is integrated between $p_0=925$ hPa and $p_1=500$ hPa. Observational reference MSE and IVT are calculated from ERA5.

Before starting the analysis, we regridded the simulations and observations to a common grid space. For the OLR, we used the NOAA CDR OLR grid spacing (with $1^{\circ }$ lat/lon). This reduces the fragmentation of OLR fields in higher-resolution simulations, resulting in a coherent structure more suitable as input into a feature-tracking algorithm. Both simulated and observational data for precipitation, circulation, and thermodynamic variables are regridded to ERA5 grid space ($0.25^{\circ }$ lat/lon). In all cases, we use the first-order conservative area-weighted regridding scheme from the “ESMF_regridding” *NCL* package (The NCAR Command Language 2023), in which each target point is calculated as the weighted mean of all input points intersecting it.

### Identification and characterisation of TE cloud band events

Tropical-extratropical cloud band events are identified through an automated detection algorithm developed by Hart et al. (2012, 2018) and adapted to SAm by Zilli and Hart (2021). The algorithm uses daily mean OLR to identify contiguous areas below a threshold indicative of deep convective cloudiness (see example in Fig. 1, shades and pink contour). To be classified as a cloud band, areas of OLR below a selected threshold should diagonally extend from the tropics to the extratropics within the region of interest (red square in Fig. 1). Here, the reference observational cloud band event set is computed independently for each model to account for unique regional domains and simulation periods. Nonetheless, the overall characteristics of these event sets are very similar to those reported in Zilli and Hart (2021).

After regridding the simulated OLR to the NOAA CDR grid, we identify the cloud band events. Positive mean OLR biases in all simulations (see Sect. 3) motivate the adjustment of the OLR threshold for each model, allowing for a fair comparison of cloud band seasonality to observations. The identification of the optimal OLR threshold follows the methodology described in Zilli et al. (2023). For each simulation, we calculate the average number of days with events and their average persistence per month, considering OLR thresholds between 210 and $275~W\,m^{-2}$ in steps of $5~W\,m^{-2}$. The difference between these values and those obtained using observed OLR (considering a $225~W\,m^{-2}$ threshold) are averaged over the rainy season (November to March—NDJFM), and the OLR threshold resulting in the smallest mean difference is chosen as the threshold for that simulation. This approach approximately constrains the season total of simulated cloud band days to the observed number. However, it places no constraint on when those days occur through the annual cycle, which is one key research focus of this study. Furthermore, it guarantees a roughly similar sample size in observations and simulations. Extensive testing with simulated and observed data demonstrated the insensitivity of results presented here to the OLR threshold.

**Fig. 2:**
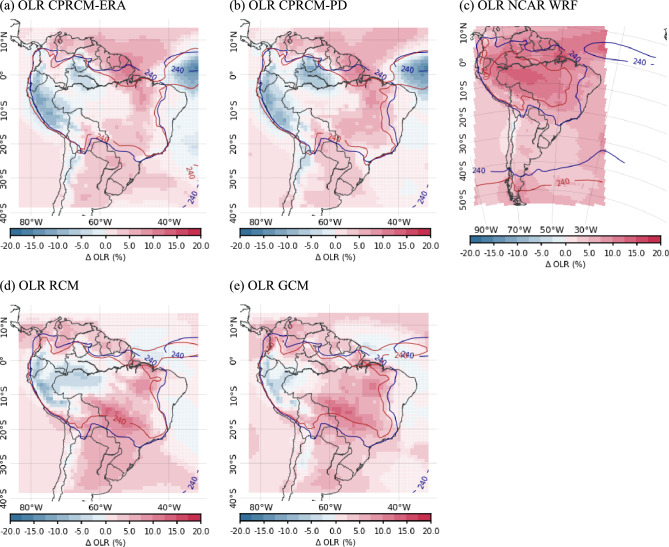
Percentage bias (shades) in the OLR climatology simulated by UKMO models (first and second columns) and by NCAR WRF (third column) during the rainy season (NDJFM), compared to observations from NOAA CDR; blue (red) contour indicating the $240~W\,m^2$ level in NOAA CDR (models). The models considered are: (**a**) UKMO CPRCM-ERA; (b) UKMO CPRCM-PD; (**c**) NCAR WRF CPRCM; (**d**) UKMO RCM; and (**e**) UKMO GCM. Note that the period of the analysis is 1998–2007 for UKMO simulations and 2000–2021 for NCAR WRF simulations

The simulated OLR thresholds that best represent the observed number of cloud band events and related persistence are $240~W\,m^{-2}$ for CPRCM-ERA, RCM, and GCM simulations. The threshold for the CPRCM-PD simulation is slightly lower, $235~W\,m^{-2}$. In the NCAR WRF CPRCM simulation, the positive bias in OLR (Fig. 2, discussed in Sect. 3) results in a higher OLR threshold of $250~W\,m^{-2}$.

To compare characteristics of rainfall during simulated and observed cloud band events, we compute the total precipitation and the fractional contribution of rainfall intensity to the total land precipitation for each day with a cloud band event, considering only values within the cloud band spatial signature (e.g. pink contour in Fig 1). The fractional contribution of rainfall rates is calculated using the precipitation distributions defined by the Analysing Scales of Precipitation (ASoP). The gridded daily precipitation is binned by intensity, with the bin rate varying in an approximately logarithmic scale (Klingaman et al. 2017), resulting in the accumulated precipitation and the number of days in each bin. The accumulated precipitation per bin is normalized by the total precipitation in each grid point, resulting in the fractional contribution of each rain rate in that season. This value is later averaged over specific sub-regions (green, blue, and orange areas in Fig. 1). All statistics are further aggregated over the days in each event before producing monthly and seasonal averages and totals.

The biases are estimated by comparing simulations to observations, with the significance estimated using a two-sided Welch’s test (Welch 1938), which is more reliable than the Student’s t-test when comparing the difference between two averages sampled from populations with unequal variance. We also compare the CPRCM results with their respective forcing models (ERA5 for NCAR-WRF) to quantify changes due to explicit convection and the effects of the lateral boundary conditions. Results are computed for the rainy season, its onset (November and December—ND) and core (January and February—JF).

## Biases in cloudiness and precipitation climatology

Before analysing the simulations of the cloud bands by the CPRCMs, we consider observed and simulated climatologies of OLR and precipitation for the rainy season (NDJFM). Halladay et al. (2023) provide a thorough analysis of the UKMO CPRCM simulations, identifying an overestimation of the mean annual precipitation over the Amazon, particularly the CPRCM-PD, and eastern Brazil, and an underestimation over the subtropics. Despite these biases, the mean precipitation simulated by the UKMO CPRCMs is lower than those in their driving simulations, particularly over northern SAm and subtropical latitudes, correcting the large wet bias in the GCM and RCM simulations compared to observations (Halladay et al. 2023).

During the rainy season (NDJFM), all UKMO simulations underestimate the OLR over the western Amazon, parts of tropical and subtropical Andes and the equatorial Atlantic Ocean, and overestimate it elsewhere in the domain (Fig. 2, first and second columns). The mean bias (spatially averaged over the entire domain) is smaller in the UKMO CPRCMs compared to their driving simulations, ranging from $+3.6~W\,m^{-2}$ for the CPRCM-PD (Fig. 2b) to $+6.8~W\,m^{-2}$ for the GCM simulation (Fig. 2e). The reduction in the positive OLR bias occurs mainly over the eastern and southern Amazon and along the SACZ. On the other hand, the magnitude of the bias in the UKMO CPRCM simulations is larger over the tropical Andes, the Atlantic ITCZ (negative biases) and off the northern coast of SAm (positive biases). The NCAR WRF CPRCM simulation overestimates the OLR over the entire domain (spatially-averaged bias of $+14.7~W\,m^{-2}$), with larger values over the Amazon and across the tropical North Atlantic Ocean (Fig. 2c). Such bias indicates the need for a different OLR threshold for cloud band detection in the WRF simulation data.

**Fig. 3:**
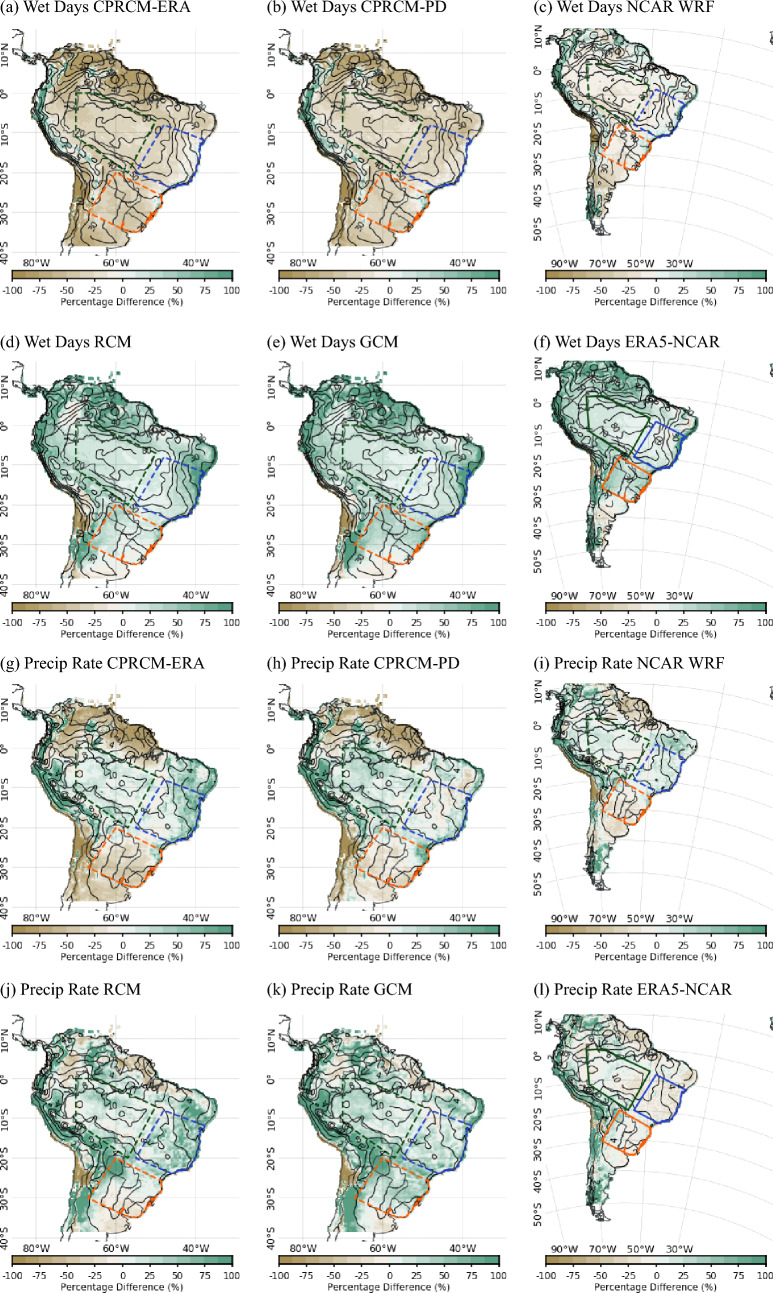
Percentage bias (shades) in the climatology simulated by UKMO models (first and second columns) and by NCAR WRF (third column) during the rainy season (NDJFM), compared to CHIRPS observational data set (contours). (**a**)–(**f**) Frequency of wet days (contours each $10\%$); and (**g**)–(**i**) Mean precipitation during wet days (contours each $2~mm\,day^{-1}$). The models considered are: (**a**), (**g**) UKMO CPRCM-ERA; (**b**), (**h**) UKMO CPRCM-PD; (**c**), (**i**) NCAR WRF; (**d**), (**j**) UKMO RCM; (**e**), (**k**) UKMO GCM; and (**f**), (**l**) ERA5-NCAR. Dashed polygons represent the southern Amazon (green), eastern Brazil (blue) and southeastern South America (orange) areas. Note that the period of the analysis is 1998–2007 for UKMO simulations and 2000–2021 for NCAR WRF simulations

**Fig. 4:**
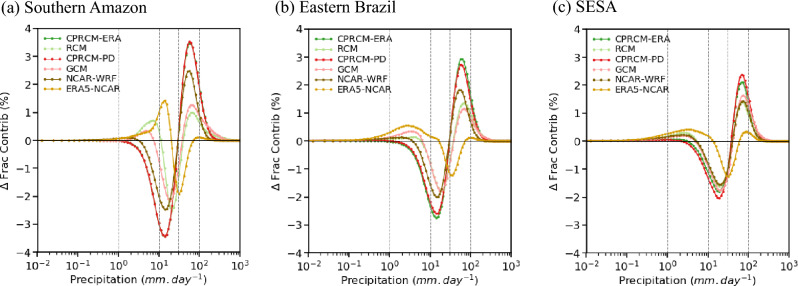
Bias in the fractional contribution (in %, y-axis) of each daily precipitation rate (x-axis) to the total rainy season (NDJFM) precipitation simulated by the models (colour key in the top left corner) compared to CHIRPS, averaged over the land areas in (**a**) southern Amazon; (**b**) eastern Brazil; and (**c**) southeastern SAm. See Fig. 1a for the location of the areas. All data sets are regridded to common $0.25^{\circ }$ lat/lon resolution. Note that the period of the analysis is 1998–2007 for UKMO simulations and 2000–2021 for NCAR WRF simulations

All UKMO CPRCMs simulate $\sim 30\%$ fewer wet days (i.e., days with precipitation above $1~mm\,day^{-1}$, considering the spatial average over the entire domain) during the rainy season compared to the observations (Fig. 3, first row, for CHIRPS; similar results when considering Br-DWGD), overcompensating the tendency of the GCM and RCM of producing a large number of days with light precipitation ($\sim 34\%$ more wet days, averaged over the entire domain; Fig. 3d and e, respectively). The NCAR WRF simulation corrects the positive bias in the number of wet days in ERA5-NCAR ($\sim 40\%$ spatially-averaged over the entire domain; Fig. 3f), resulting in $\sim 6\%$ fewer wet days compared to the observations (Fig. 3c). Note that here “ERA5-NCAR” indicates the ERA5 as the “driving model” forcing the NCAR WRF simulations. The overestimation of light precipitation rates and drizzle (precipitation below $1~mm\,day^{-1}$) by the driving simulations (RCM, GCM, and ERA5-NCAR) is evident when analysing the contribution of rain rates to the total precipitation, especially over Southern Amazon (SAmz) and Eastern Brazil (EBr; Fig. 4a and b; see Fig. 3 for the location of these areas). Improvements in the representation of wet day frequency by CPRCM simulations are also observed in other regions (Lucas-Picher et al. 2021), particularly over West Africa using a similar UKMO CPRCM configuration (Berthou et al. 2019).

The bias in the precipitation during wet days, i.e. rain rate, is positive in the UKMO CPRCMs (spatially-averaged bias of $4\%$ and $10\%$ in CPRCM-ERA and CPRCM-PD, respectively), but smaller than in their driving simulations ($\sim 35\%$ in the RCM and GCM; Fig. 3g, h, j, and k). The reduction in the average bias in the CPRCM simulations arises from a reduction in the magnitude of the wet bias over Amazon and Eastern Brazil combined with dry biases over the northern and subtropical SAm, not present in the driving simulations. In all CPRCM simulations, the wet bias is driven mainly by a larger contribution of heavy precipitation rates ($\ge 30~mm\,day^{-1}$) at the expense of light precipitation rates ($1-10~mm\,day^{-1}$), regardless of the observational data sets (Fig. 4 for CHIRPS; Br-DWGD not shown). The same tendency also occurs at the sub-daily scales (Halladay et al. 2023). Over the tropics, the biases in rain rates simulated by the CPRCMs are larger than in their driving simulations (Fig. 4a and b), while over Southeastern SAm (SESA; orange area in Fig. 3), both CPRCMs and driving simulations have similar biases (Fig. 4c). The NCAR WRF simulation has a similar spatially-averaged bias in precipitation rate as ERA5-NCAR driving simulation (Fig. 2i and l), even though this simulation also overestimates (underestimates) the contribution of heavy (light) rain rates (Fig. 4).

## Simulated cloud band event frequency and precipitation characteristics

**Fig. 5:**
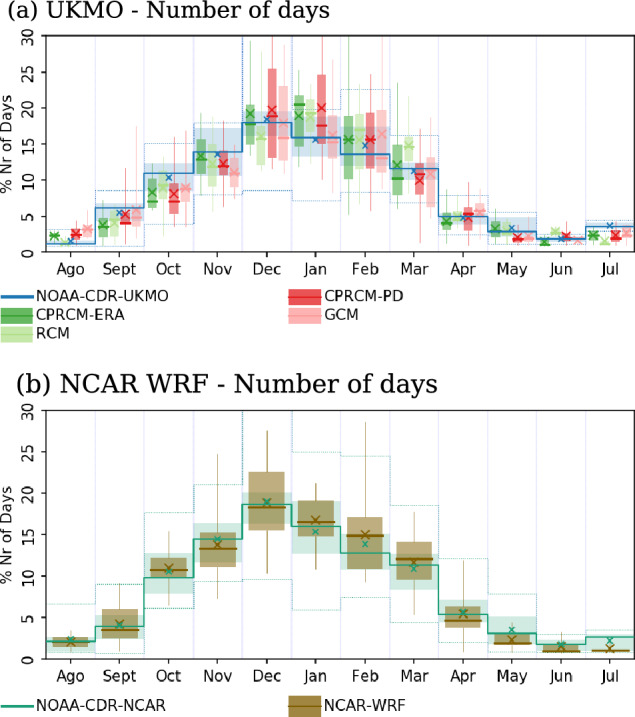
Number of days with cloud band events per month over the region of interest (red square in Fig. 1), expressed as the percentage of the total annual number of days with events, considering (**a**) UKMO and (**b**) NCAR WRF simulations. Simulations (colour legends shown at bottom) represented by boxplots (monthly median and interquartile range) and whiskers (minimum and maximum values) are compared to observed values obtained using NOAA CDR OLR, represented by the dark blue line (monthly average), shades (interquartile range), and dotted lines (minimum and maximum values). In all data sets, the crosses represent the mean percentage number of days per month. Period of the analysis: 1998–2007 for UKMO simulations and 2000–2021 for NCAR WRF simulations

All simulations reproduce the observed annual cycle of the cloud band days (Fig. 5) to within $\sim$30% of observations, with a maximum percentage of the days with events during the rainy season (NDJFM) and a minimum during austral winter (JJA). Nonetheless, the UKMO simulations underestimate the number of cloud band days between September and November (Fig. 5a), with improvements in the CPRCMs compared to their driving simulations. During the austral summer (DJF), the simulated events are longer, resulting in a $\sim$25% overestimation of the number of days with events in all UKMO simulations, with no clear improvement by the CPRCMs. NCAR WRF simulated the annual cycle to within $\sim$10% of observed cloud band days by month (Fig. 5b). Note that the analysis period and the study area are slightly different between UKMO and NCAR WRF simulations, resulting in different numbers of days with observed cloud bands for each set of simulations.

These results show distinct biases, particularly in the UKMO CPRCM simulations, during the rainy season’s onset (ND) and core (JF). Thus, we will analyse the intensity and frequency of cloud bands during these two seasons separately in the following sections.

### Cloud band simulation during rainy season onset

**Fig. 6:**
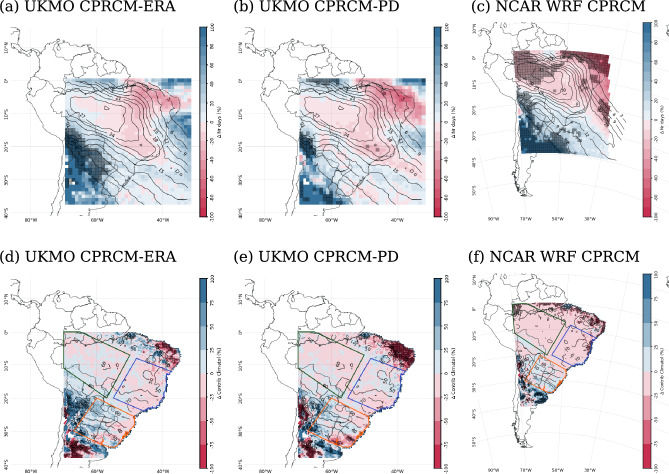
Percentage biases (shades) in simulated cloud band events during the onset of the rainy season (ND), compared to observations (contours): (**a**)–(**c**) number of days with events (contours each $3~days~per~2~months$), with the observational data set from NOAA CDR and simulated data sets regridded to $1^{\circ }$ lat/lon. (**d**)–(**f**) Percentage contribution of the cloud band events to the total precipitation (contours each $10\%$), with the observational data set from CHIRPS; both observational and simulated data sets are regridded to $0.25^{\circ }$ lat/lon. In all maps, simulations are from UKMO CPRCM-ERA (first column), UKMO CPRCM-PD (second column), and NCAR WRF CPRCM (third column). Areas where the bias is statistically significant ($p<0.05$) are shaded grey. Note that the period of the analysis is 1998–2007 for UKMO simulations and 2000–2021 for NCAR WRF simulations

During the onset of the rainy season (ND), all CPRCM simulations place the maximum in the number of days with cloud bands over the Amazon and across Southeastern Brazil into the adjacent South Atlantic Ocean (e.g., Fig. 6a–c). Significant biases occur over the southwestern portion of the domain, where the small number of observed events (less than one day per season on average) results in a large percentage increase in cloud band days in the models.

Over tropical latitudes, the UKMO CPRCM simulations shown in Fig. 6 reduce the magnitude of the biases in their driving simulations (not shown) and improve the representation of the cloud band’s contribution to the total precipitation. In fact, the bias in the contribution of the simulated cloud bands to the climatology is within $\pm 20\%$ over most of the tropics (Fig. 6d, e), indicating that the UKMO CPRCM simulations correctly represent the importance of the cloud bands to the seasonal precipitation. Over SAmz, the bias in the number of days with events simulated by the UKMO CPRCMs is small ($<\pm 10\%$; Fig. 6a and b). Over EBr, the bias is larger ($\sim -10\%$), resulting in fewer days with events ($\sim -2$ days per 2 months). The largest negative bias in the number of days with events occurs over the same region where the bias in the OLR climatology is positive, i.e. too little deep cloud (Fig. 2). Over SESA, the UKMO CPRCMs simulate $20-30\%$ more days with cloud band events, with fewer events in the CPRCM-PD compared to CPRCM-ERA, despite the CPRCM-PD being the wettest simulation (Halladay et al. 2023). The NCAR-WRF simulation also underestimates the number of days with cloud band events over EBr and SAmz ($15\%$ fewer days; Fig. 6c) and overestimates it over SESA ($30\%$ more days).

To assess how cloud bands affect the structure of the precipitation distribution and how these effects are (better) captured by the convective-permitting simulations, we compare the total precipitation, the anomalies in the precipitation during wet days ($PP~\ge ~1~mm\,day^{-1}$) and the anomalies in the fractional contribution of rain rates to total precipitation during cloud bands events to the observational data sets. Due to the similarity between the CPRCM-ERA and CPRCM-PD simulations, we will only present the results for the CPRCM-ERA simulations. Whenever necessary, we comment on the relevant differences between the simulations.

**Fig. 7:**
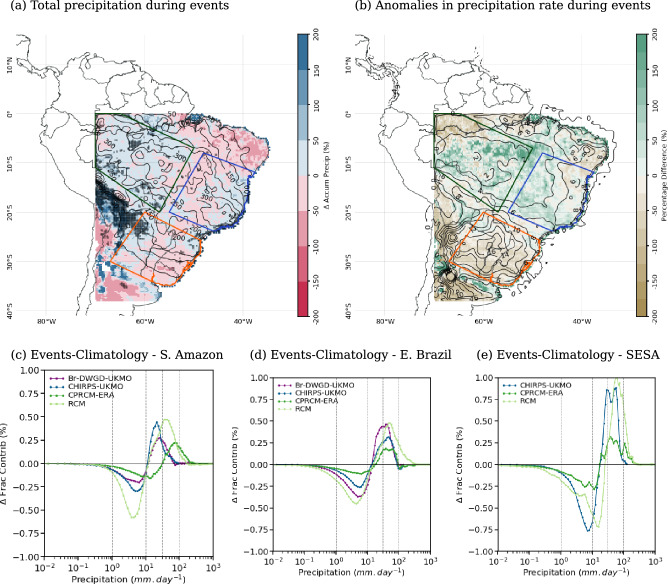
(**a**), (**b**) Percentage biases (shades) in the cloud band events during the onset of the rainy season (ND) simulated by the UKMO CPRCM-ERA compared to CHIRPS (contours): (**a**) total precipitation (contours each $50~mm~per~2~months$); and (**b**) anomalies in precipitation rate during wet days (contours each $2~mm\,day^{-1}$, with negative values dashed). Areas where the bias is statistically significant ($p~<~0.05$) are shaded grey. (**c**)–(**e**) Difference in the fractional contribution (in %, y-axis) of each daily precipitation rate (x-axis) between the days with cloud band events and the climatology in the CPRCM-ERA and RCM simulations, CHIRPS, and Br-DWGD (except in (**e**)) considering the onset of the rainy season (ND). The legend for each data set is shown in the top left corner. All data sets are regridded to $0.25^{\circ }$ lat/lon resolution. Values are averaged over the land areas (**c**) southern Amazon; (**d**) eastern Brazil; and (**e**) southeastern South America. See Fig. 1a for the location of the areas. Note that the period of the analysis is 1998–2007 for UKMO simulations and 2000–2021 for NCAR WRF simulations

**Fig. 8:**
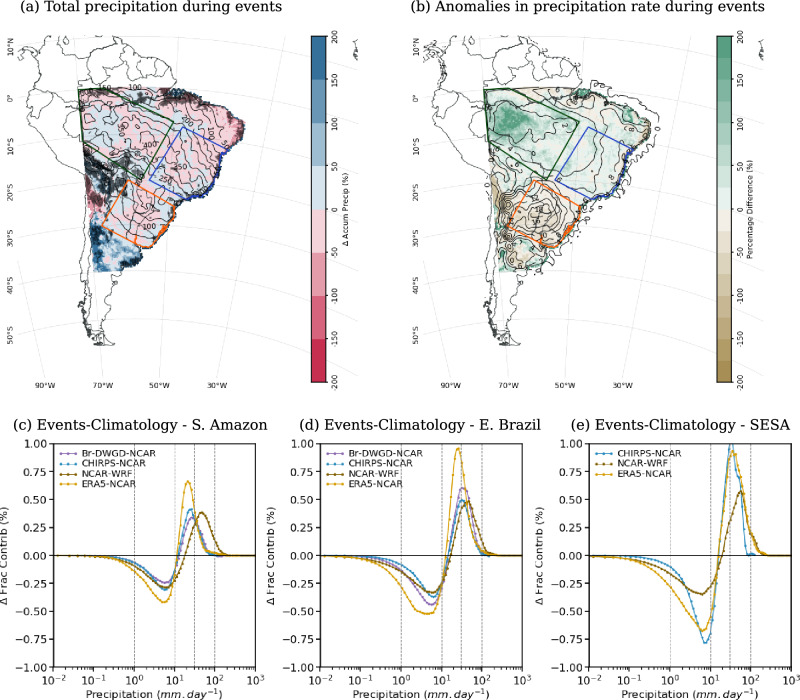
As in Fig. 7, comparing the NCAR WRF CPRCM simulation to CHIRPS-NCAR

In observational data sets, the presence of cloud bands results in an enhancement of precipitation by more than $10~mm\,day^{-1}$ above climatology over most parts of SESA (contours in Fig. 7b). In the CPRCMs simulations (both UKMO and NCAR-WRF), the precipitation anomalies are $\sim 20\%$ smaller over SESA (Figs. 7b and  8b). Over SAmz and EBr, the observed anomalies in precipitation rate are smaller ($<2~mm.day^{-1}$ and $<8~mm.day^{-1}$, respectively), with the simulations overestimating them by more than $10\%$ (Figs. 7b and  8b).

Over all regions, the presence of observed cloud bands shifts the distribution of the fractional contribution of precipitation rates to the right, resulting in an increase (reduction) in the contribution of rain rates above (below) $10~mm\,day^{-1}$ (Figs. 7c–e and 8c–e). All CPRCMs reproduce this shift, albeit with muted magnitudes. Over SAmz, the presence of cloud bands in the CPRCMs simulations increases the contribution of rain rates above $30~mm.day^{-1}$ (Figs. 7c and 8c), increasing the precipitation by $15\%$ or more (Figs. 7b and 8b). This increase in the precipitation rate occurs during fewer events but still results in an increase in total precipitation during simulated cloud bands (Figs. 7a and 8a), more evident in the UKMO simulations. Despite overestimating the shift toward higher rain rates, the CPRCMs better represent the changes in the fractional contribution compared to their forcing models, improving the simulations of the precipitation during cloud band events over the region.

Over EBr, the contribution of rain rates below $10~mm\,day^{-1}$ is larger in the UKMO CPRCMs compared to observations, resulting in precipitation rates 20–30% larger over the region (Fig. 7b). However, this excessive precipitation rate is distributed during fewer cloud band events, resulting in a small bias in the total precipitation (Fig. 7a). Compared to their forcing simulation, the UKMO CPRCMs reduce the bias in the precipitation rate (not shown), indicating some improvement due to explicitly resolving the convection over the region. The NCAR WRF correctly simulate the shift in the fractional contribution of the precipitation (Fig. 8d) but overestimates the precipitation rate in $\sim 10\%$ (Fig. 8b). Nonetheless, the reduction in the number of events results in negative bias in total precipitation (Fig. 8a) and $-13\%$ reduction in the contribution of the events to the climatology (Fig. 6f).

Over SESA, all CPRCMs underestimate the contribution of rain rates above $20~mm\,day^{-1}$ (Figs. 7e and 8e), resulting in negative biases in precipitation rate ($\sim -20\%$, Figs. 7b and 8b). Note that the comparison with the Br-DWGD data set is not available for this region because the data set only covers the Brazilian part of the area. The increased number of days with cloud band events simulated by the CPRCMs compensates for the negative bias in the precipitation rate, resulting in a correct simulation of the total precipitation during cloud band events (Figs. 7a and 8a).

### Cloud band simulation during the core rainfall season

**Fig. 9:**
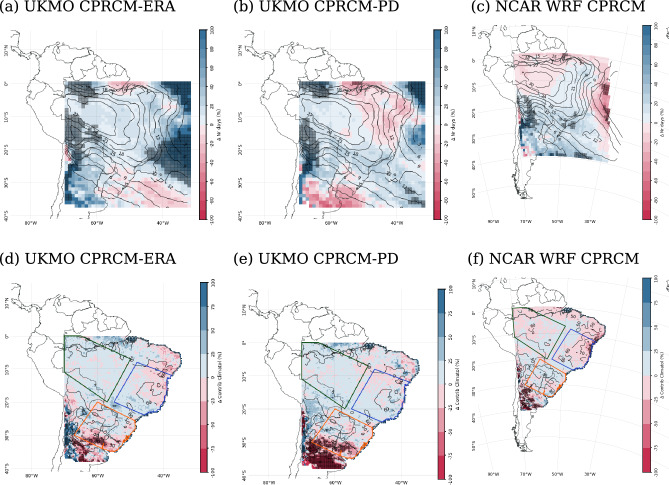
As in Fig. 6, for the core of the rainy season (JF)

During the core of the rainy season (JF), the UKMO CPRCMs simulate $\sim 20\%$ more cloud band days, particularly over western Amazon and between $20^{\circ }S$ and $25^{\circ }S$ (Fig. 9a and b), and indeed more cloud-band days than simulated in their driving models (not shown). Despite that, the bias in the contribution of cloud bands to the rainfall climatology is within $\pm 10\%$ of the observations over most parts of the tropics (Fig. 9d–f). Additionally, the UKMO CPRCM-ERA simulates more events north of $20^{\circ }S$ and east of $50^{\circ }W$ (Fig. 9a), shifting the mean location of the cloud bands northeastward, while the UKMO CPRCM-PD has fewer cloud band days over the same region (Fig. 9b). In contrast, over the subtropics, the biases in the number of cloud band events simulated by the UKMO CPRCMs are also positive ($\sim +10\%$) but with a reduced contribution to the rainfall climatology ($\sim -10\%$). NCAR WRF CPRCM simulates fewer days with cloud bands over the Amazon and more days with cloud bands over EBr and south of $\sim 20^{\circ }S$ (Fig. 9c).

**Fig. 10:**
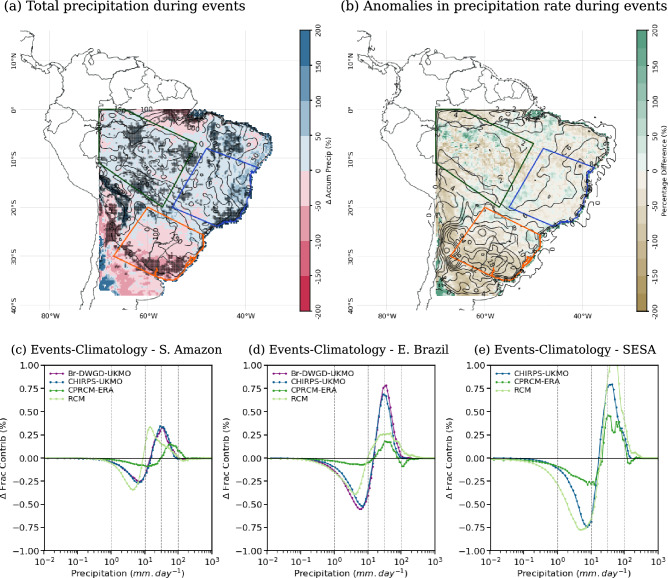
As in Fig. 7, but considering the core of the rainy season (JF)

**Fig. 11:**
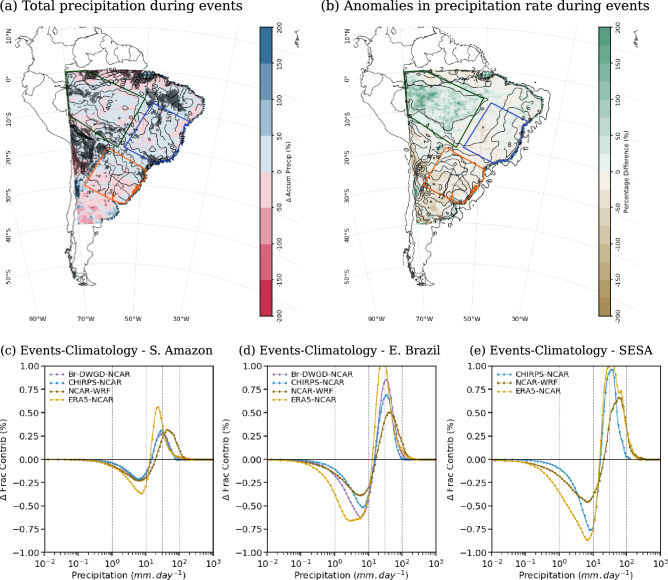
As in Fig. 7, but considering the NCAR WRF CPRCM simulation during the core of the rainy season (JF)

In this season, the presence of cloud bands increases the observed precipitation anomalies south of $20^{\circ }S$ (black contours in Fig. 10b) and, as observed during the onset, shifts the precipitation distribution towards higher rain rates (Figs. 10c–e and 11c–e). Over the SAmz, the increase in the contribution of rain rates above $20~mm\,day^{-1}$ compensates for the decrease in lower rain rates (see purple and dark grey lines in Fig. 10c), resulting in precipitation rate anomalies below $2~mm.day^{-1}$ over the region (black contours in Fig. 10b). In all CPRCMs, the shift is correctly simulated, but its magnitude is underestimated (Figs. 10c–e and 11c–e).

As observed by Halladay et al. (2023), the UKMO CPRCM-PD (and the GCM) simulation is wetter than the CPRCM-ERA (and RCM). This difference is also evident when comparing the precipitation rate anomalies during simulated cloud band events. Over SAmz and EBr, both simulations have areas with positive and negative biases, with the CPRCM-ERA showing predominantly negative biases in relation to CHIRPS observations ($-13\%$ and $-8\%$, respectively; Fig. 10b) while in the CPRCM-PD simulations the bias is mostly positive ($7\%$ and $13\%$, respectively; not shown). Compared to Br-DWGD, the bias in the precipitation rate is positive in both simulations ($>20\%$, not shown). Regardless of the observational data set, the UKMO CPRCMs overestimate the contribution of rain rates above $40~mm\,day^{-1}$ but underestimate it between 10 and $40~mm\,day^{-1}$ over SAmz (Fig. 10c). Combined with more frequent cloud bands, this results in $\sim 25\%$ more precipitation during the events (Fig. 10a), increasing their contribution to the climatology by $\sim 10\%$ (Fig. 9d and e).

The NCAR-WRF simulation also overestimates the contribution of heavier rain rates (Fig. 11c), resulting in precipitation rate anomalies $55\%$ more intense (compared to CHIRPS) over SAmz (Fig. 11b). The more intense precipitation rates are distributed over fewer cloud band days (Fig. 9c), resulting in the correct amount of precipitation (Fig. 11a) and its contribution to the precipitation during JF (Fig. 9f). Over this region, explicitly simulating the convection does not improve the representation of the distribution of rain rates during cloud bands despite smaller biases in the UKMO CPRCMs than in their driving simulations (not shown).

Over EBr, all CPRCMs correctly increase (reduce) the fractional contribution of rain rates above (below) $20~mm\,day^{-1}$, better represented in the NCAR WRF simulation (Figs. 10d and  11d). With that, the precipitation rate anomalies during simulated cloud bands are within $\pm 10\%$ the observed values (Figs. 10b and 11b). Nonetheless, cloud band events are too frequent in these simulations, resulting in a wet bias ($>10\%$) during cloud band events over EBr (Fig. 10a and Fig. 11a).

Over SESA, all simulations have opposite biases to the tropics, underestimating the precipitation rate anomalies by $-15\%$ or more (Figs. 10b and 11b), with the UKMO CPRCM simulations drier than their driving model (figure not shown). As a result, the biases in the total precipitation over the region are negative (Figs. 10a and  11a) despite the larger frequency of simulated cloud bands (Fig 9a–c).

## Interactions between the thermodynamic and dynamic mean state and the synoptic scales

All UKMO CPRCM simulations have biases in the frequency and intensity of the cloud band events. Throughout the rainy season, the cloud band events simulated by the UKMO CPRCMs are $\sim -15\%$ less intense but 10–30% more frequent than the observations in the subtropics. In the tropics, the simulated events are at least $10\%$ as intense than the observations during the entire rainy season, but $\sim 10\%$ less frequent than observed during the onset (ND), particularly over EBr, and $\sim$20% more frequent during the core (JF) season. This suggests that part of the bias during the synoptic scale simulated events is related to biases in the simulated basic state interacting with the anomalies during cloud band events. To investigate the impacts of the simulated basic state on the formation of the cloud bands, we analyse the biases in the climatology of the moist static energy (MSE) and integrated water vapour transport (IVT), presented in the next subsections. The implications of these biases to the formation and persistence of cloud band events are discussed in Sect. 6. Here, the simulations are compared to the ERA5 reanalysis, which is also the lateral boundary conditions in the NCAR WRF simulation. Thus, to maintain a fair evaluation of the simulations using independent sources, we focus only on the UKMO CPRCM simulations.

### Biases in the upper-level circulation

**Fig. 12:**
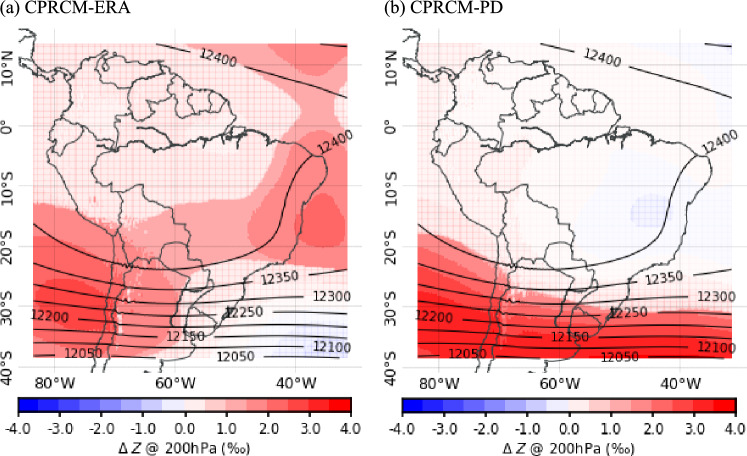
Per mille biases (‰; shades) in the upper-level (200 hPa) geopotential height climatology during the rainy season (NDJFM) simulated by (**a**) CPRCM-ERA and (**b**) CPRCM-PD, compared to ERA5 observations (contours each 50 *m*). Models regridded to ERA5 grid space. Areas with non-significant bias ($p>0.05$) are whited out

As demonstrated by Zilli et al. (2023), the biases in the frequency of cloud bands in the HadGEM3-GC3.1 simulations are related to biases in the large-scale upper-level circulation over the extratropics. During the rainy season (NDJFM), the UKMO simulations considered here also show small (within 3‰) but significant biases in the upper-level circulation south of $20^{\circ }S$ over subtropical eastern Pacific (Fig. 12 for CPRCM-ERA and CPRCM-PD).

In the CPRCM-ERA simulation (and its driving model), the bias in the 200 hPa geopotential height in the NDJFM basic state is 2‰positive (1‰negative) over the tropical (subtropical) South Atlantic Ocean (Fig. 12a). This bias results in a small equatorward shift of the upper-level trough over the subtropical western South Atlantic Ocean, increasing the westerly winds over subtropical latitudes ($\sim 25^{\circ }S$). The CPRCM-PD and the GCM simulate an opposite bias to the ERA-driven simulation in upper-level basic states, overestimating the geopotential height south of $\sim 25^{\circ }S$ (> 4‰) and underestimating it over EBr and the adjacent tropical South Atlantic Ocean, albeit the latter with muted intensity (Fig. 12b for CPRCM-PD; GCM not shown).

### Biases in the MSE and IVT

**Fig. 13:**
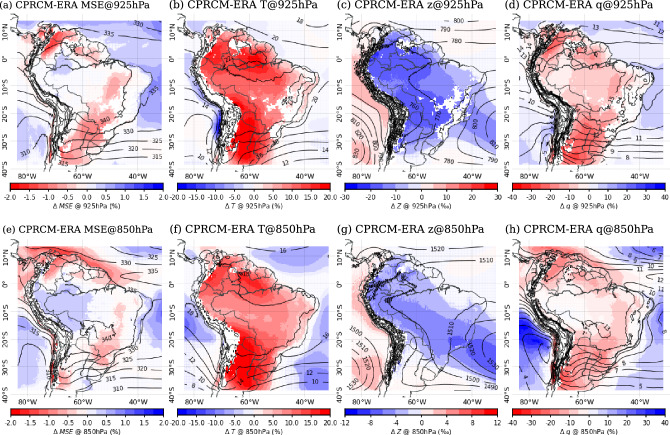
Percentage biases (per mille ‰ in (**c**) and (**g**); shades) in the CPRCM-ERA climatology during the rainy season (NDJFM), compared to ERA5 observations (contours): (**a**) and (**e**) MSE (contours each $5~kJ\,kg^{-1}$); (b) and (f) temperature (contours each $2^{\circ }C$); (**c**) and (**g**) geopotential height (contours each 10 *m*); and (**d**) and (**h**) specific humidity (contours each $1~g\,kg^{-1}$). Values are calculated at (**a**)–(**d**) 925 hPa and (**e**)–(**h**) 850 hPa. Models are regridded to ERA5 grid. Areas with non-significant bias ($p>0.05$) are whited out

The climatological specific humidity of the UKMO CPRCM simulations is drier than their driving models (not shown) at lower levels (925 hPa and 850 hPa), resulting in lower values of MSE over most of the domain. As a result, the CPRCMs better represent the observed MSE climatology over the Amazon region, where the bias is small but positive, but degrade it over parts of EBr and SESA, resulting in negative bias over these regions (Fig. 13a and e). Furthermore, the differences with respect to the driving simulations suggest that the biases in the MSE basic state of the CPRCMs are driven mainly by explicitly resolving convection. However, as will be discussed next, the small MSE biases (± 2%) are underpinned by larger (± 20%) biases in the MSE constituent terms with warm temperatures counteracting lower specific humidities.

**Fig. 14:**
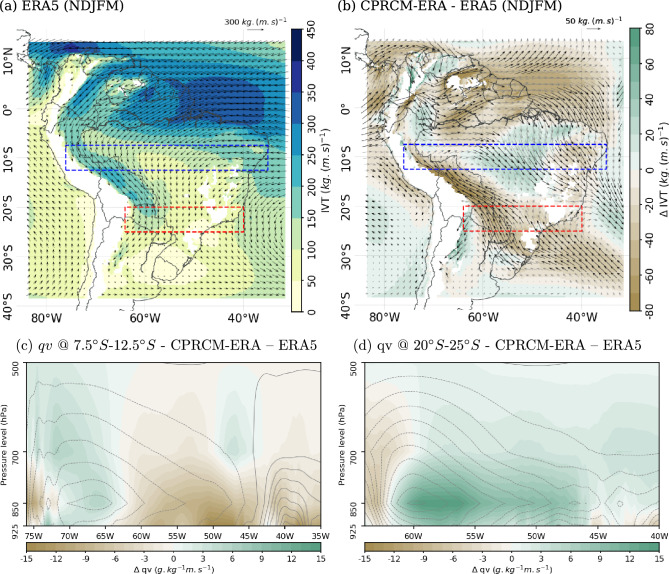
(a)–(b) IVT integrated between 925 hPa and 500 hPa during the rainy season (NDJFM). (**a**) ERA5 climatology (shades and arrows in $kg\,(m\,s)^{-1}$). (**b**) Bias in the CPRCM-ERA simulation compared to ERA5 (shades and arrows, in $kg\,(m\,s)^{-1}$). In (**b**), black arrows indicate areas with significant changes in the zonal and meridional components of the IVT (p<0.1). (**c**)–(**d**) Bias in the meridional water vapour transport between 925 hPa and 500 hPa (*vq*, in $(g\,kg^{-1})(m\,s^{-1})$), averaged over (**c**) $7.5^{\circ }S$–$12.5^{\circ }S$ (dashed blue box in (**a**) and (**b**)) and (**d**) $20^{\circ }S-25^{\circ }S$ (dashed red box in (**a**) and (**b**)). In (**c**) and (**d**), the contours represent the meridional moisture transport climatology in ERA5 (every $5~(g\,kg^{-1})(m\,s^{-1})$; northerly dashed). Models regridded to ERA5 grid space

Over EBr, the negative bias in the simulated near-surface MSE basic state results mainly from lower values of specific humidity in the CPRCM simulations (Fig. 13d and h). There are also biases in the other terms of MSE (Eq. 1), with positive biases in temperature (Fig. 13b and f) and negative biases in geopotential height (Fig. 13c and g). The reduced values of specific humidity are likely the result of weaker IVT along the northern coast, reducing the moisture transport into the continent by the trade winds (Fig. 14b). Furthermore, the northwesterly bias over Northeast Brazil results in a stronger northerly moisture transport below 850 hPa (Fig. 14c). With that, more moisture is transported southward, resulting in a drier lower troposphere over the region. Similar but slightly weaker biases in IVT also occur in the driving simulations (not shown). Combined with warmer temperatures, these biases suggest an increase in the moisture required by the CPRCM to reach saturation and support deep convection over the region in the simulated basic state.

During synoptic-scale cloud band events, the anomalous lower- and mid-level cyclonic circulation increases the westerly transport of moisture from the Amazon into eastern Brazil (see Zilli and Hart 2021; Zilli et al. 2023 for examples of the circulation anomalies during cloud band events). Thus, when the synoptic disturbances associated with cloud bands are strong enough, the water vapour advected into the region maintains the convection characteristic of the events.

Over SAmz, the UKMO CPRCMs simulate positive biases in the lower-level MSE basic state (Fig. 13a and e). At 925 hPa, the UKMO CPRCMs simulations are warmer and drier than the observations (Fig. 13b and d, respectively), compensating one another together with negative biases in geopotential height (Fig. 13c), which results in small MSE biases. At 850 hPa, the dry bias in the specific humidity climatology is reduced (Fig. 13h), resulting in a positive bias in the MSE (Fig. 13e). The bias in the IVT suggests that this region is drier due to a reduction in the moisture transported from the tropical North Atlantic Ocean by the trade winds (Fig. 14b). Additionally, the UKMO CPRCMs simulate a weaker basic state northwesterly IVT from the Amazon (Fig. 14b), suggesting a reduction in moisture transported out of the region by the LLJ (Fig. 14c). This reduction could also be related to the smaller negative bias at 850 hPa compared to lower levels (Fig. 13h).

Over the Amazon, cloud bands do not significantly modify the basic state due to the intense convective activity already present throughout the rainy season. The UKMO CPRCMs and their driving models correctly represent this behaviour (not shown).

In the UKMO CPRCM simulations, the reduction in the basic state northwesterly IVT from the Amazon (Fig. 14b) weakens the meridional moisture transport at lower levels (Fig. 14d), contributing to the reduction in specific humidity over subtropical SAm (Fig. 13d and h). In the driving simulation, the northwesterly IVT is stronger than in ERA5, resulting in larger precipitation rates over the region (not shown). During UKMO CPRCM simulated cloud band events, lower-level cyclonic anomalies (figure not shown; see Zilli and Hart 2021 for examples of the circulation anomalies during cloud band events) weaken the northerly flow at their western flank, reducing the moisture advected towards the region. Combined with a drier basic state, these biases result in less intense cloud band events in the UKMO CPRCM simulations over the region (Fig. 7b).

## Discussions and conclusion

Previous studies demonstrated the added value of using CPRCMs to simulate extreme precipitation events in daily and sub-daily scales (Kendon et al. 2019; Halladay et al. 2023). Hart et al. (2018) also showed how a large-domain convective-permitting simulation can overcome poor driving model boundary conditions to vastly improve the annual cycle of tropical-extratropical cloud band events over Southern Africa. Here, we show that the UKMO and the NCAR WRF CPRCMs simulate the frequency and the seasonal cycle of the tropical-extratropical cloud band events over SAm to within 10–30% of observations. For the UKMO CPRCM simulations, this improves upon the driving simulation mainly during the onset of the rainy season (ND). During cloud band events, the CPRCM simulations captured the observed shift of the precipitation distribution toward higher rain rates, including when compared to the gridded rain gauge precipitation data set (Br-DWGD). Furthermore, the CPRCM models also reproduce the location and intensity of the circulation and thermodynamic anomalies observed during cloud bands.

**Fig. 15:**
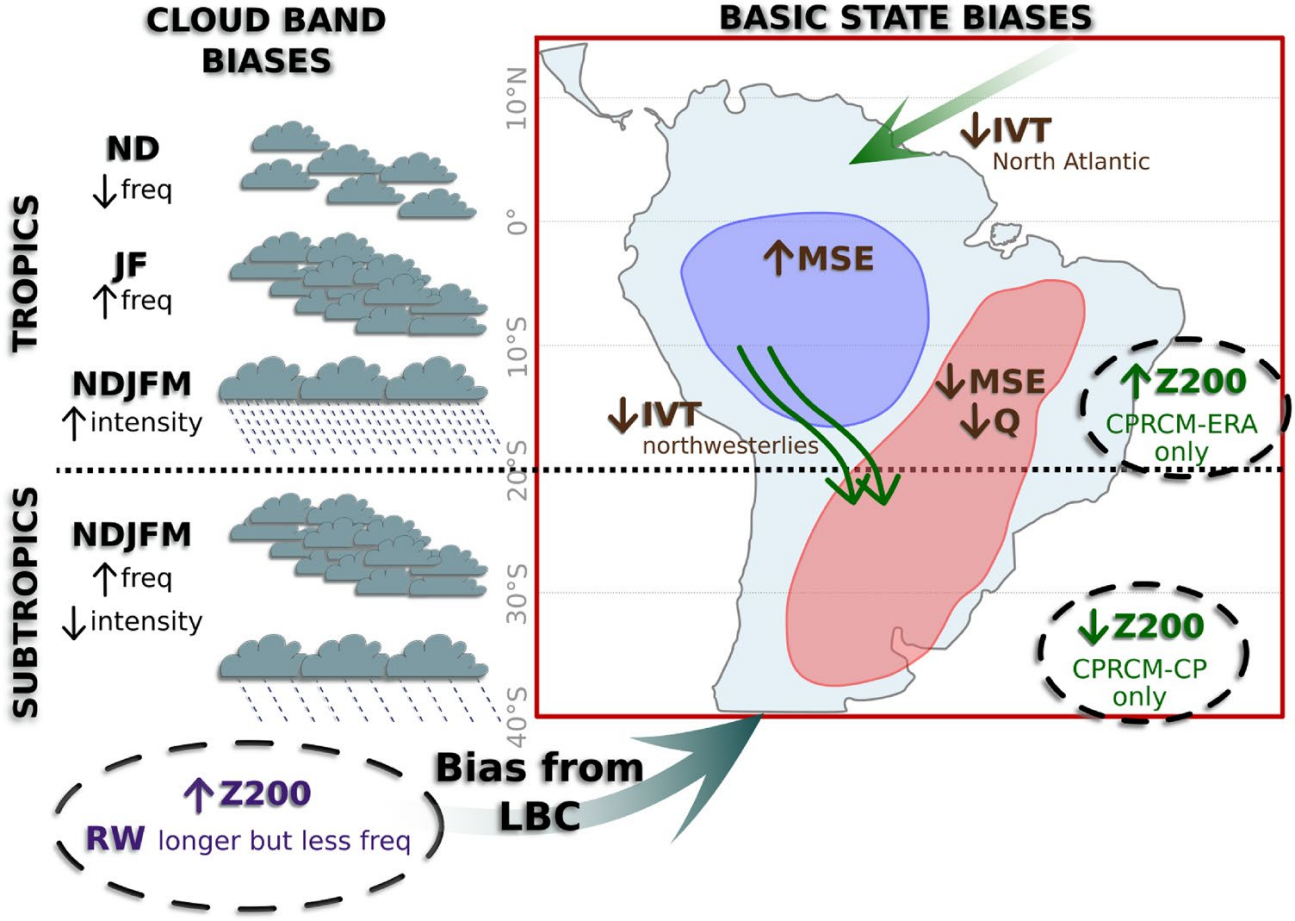
Schematic summarising the main biases during cloud bands (left) and in the basic state (right). Bias in the frequency of cloud band events (represented as the number of clouds) and precipitation intensity during cloud bands. The upward (downward) arrows indicate a positive (negative) bias in the main state. Text colours indicate biases from the lateral boundary conditions (purple), biases with similar magnitude in the CPRCMs and their forcing models (green), and biases present only in the CPRCMs (brown). See text for more details

Despite improvements, the CPRCM simulations have biases in the frequency of cloud band events and their precipitation intensity (represented in Fig. 15) that affect the contribution of these events to the rainfall climatology. In all CPRCM simulations, the cloud band events are too frequent during the core of the rainy season. During the onset of the rainy season, the CPRCMs simulate fewer events than observed over Eastern Brazil but too many events over the subtropics (Fig. 15, left). There is also a clear geographical distinction regarding the bias in the intensity of the events, with the CPRCMs overestimating the precipitation over the tropics but underestimating it over the subtropics (Fig. 15, left). Most of the biases related to the frequency of events are caused by biases in the lateral boundary conditions from the driving models combined with local biases in the mean state simulated by the CPRCMs.

Most biases related to the frequency of the cloud band events simulated by the CPRCMs can be explained by small biases in the upper-level circulation. In the UKMO CPRCM-PD and GCM simulations, the positive bias in upper-level geopotential height south of $30^{\circ }S$ (represented as “Z200” over mid-latitude South Atlantic in Fig. 15, left) changes the tropical-extratropical zonal wind shear along the eastern Brazilian coast, hindering the propagation of mid-latitude disturbances into tropical latitudes but increasing the number of events over SESA. This bias is stronger during the onset of the rainy season (ND), a transition period with large variability. Zilli et al. (2023) also identified similar biases in the cloud band events simulated by the HadGEM3-GC3.1, which has the same model dynamical core as the UKMO CPRCMs. In the CPRCM-ERA and RCM simulations, the upper-level geopotential height bias is the opposite (“Z200” over tropical South Atlantic in Fig. 15, left), increasing the upper-level westerlies over the subtropical South American coast ($\sim 25^{\circ }S$) and favouring the propagation of Rossby Wave disturbances deeper into tropical latitudes. This mechanism is predominant during JF, when the CPRCM-ERA simulates too many cloud band events over Northeastern Brazil. These biases in geopotential height are also present in the driving models, indicating that they are likely related to the lateral boundary conditions.

Zilli et al. (2023) demonstrated that biases in the intensity of the mid-latitude upper-level westerlies over the central subtropical South Pacific change the spectrum of the Rossby waves reaching SAm, reducing the frequency of cloud band events but increasing their duration in the HadGEM3-GC3.1 simulations. The simulations considered by those authors are very similar to the driving GCM simulation for CPRCM-PD. Thus, it is safe to assume that part of the bias in the number of cloud band events in the CPRCM-PD is inherited from the biases in the GCM large-scale circulation (purple “Z200” and “RW longer but less freq” in Fig. 15). Furthermore, the dynamical core of the RCM simulations is the same as in the GCM and the HadGEM3-GC3.1 simulations in Zilli et al. (2023). Thus, part of the biases in the frequency of cloud band events in the CPRCM-ERA simulations may result from similar large-scale circulation biases in the RCM, despite the RCM being forced by more realistic lateral boundary conditions from ERA-Interim reanalysis.

Locally, the large-scale bias in the circulation interacts with the biases in the MSE. Over EBr, the reduction in the MSE in the basic state (“MSE” over Eastern Brazil in Fig. 15) is related to weaker trade winds, reducing the moisture transport into the region (“IVT North Atlantic” in Fig. 15) and drying atmosphere at lower levels (“Q” in Fig. 15), thereby lowering energy available to support deep convection and affecting the spatial coherence necessary to form a continuous cloud band over the region, resulting in fewer events. This bias occurs throughout the rainy season and is more evident in the CPRCM-PD simulations. In the CPRCM-ERA simulation, the reduced MSE is stronger during the onset of the rainy season (ND); in JF, the bias in the upper-level geopotential height prevails, resulting in a larger number of cloud band events over the region.

Over the Amazon, the small basic state MSE bias masks significant dry biases in specific humidity at 925 hPa. At 850 hPa, the dry bias is reduced due to weaker northwesterly IVT, reducing the moisture transport out of the region (“IVT northwesterlies” in Fig. 15) and resulting in small positive biases in the MSE (“MSE” over the Amazon in Fig. 15). The reduction in the magnitude of the positive MSE and specific humidity biases from the driving simulation to the CPRCMs with respect to the observations suggests that the CPRCMs are more efficient in consuming the available energy and producing precipitation.

In both these regions, the biases in the basic state IVT help explain the biases in the frequency of cloud bands simulated by the UKMO CPRCMs but are not enough to justify the intense precipitation rates both in the climatology and during cloud band events. Compared to their driving simulations, the MSE bias in the UKMO CPRCMs is reduced due to a reduction in the specific humidity bias, whereas it is similar for the other terms. This suggests that CPRCMs are more efficient in consuming energy and producing precipitation than their driving parametrised models. Thus, biases in the precipitation intensity are likely associated with explicitly resolving the convection and the interplay with the model’s configurations and other parametrisations, such as cloud microphysics schemes, as suggested by Halladay et al. (2023).

The bias in the NCAR WRF CPRCM simulations corroborates the hypothesis about the origin of precipitation intensity biases. The NCAR WRF CPRCM has a different dynamical core and is forced by different lateral boundary conditions (ERA5). Nonetheless, it also overestimates the precipitation rates in the basic state and during cloud bands, with spatial patterns similar to those observed in the UKMO CPRCM simulations. Thus, the next steps in evaluating the quality of the CPRCM simulations should explore the consequences of explicitly resolving the convection, including the relationship with sub-grid scale convective processes not resolved at this resolution and land-atmosphere coupling.

Over SESA, the simulated intensity of the cloud bands is also affected by weaker northwesterly IVT (“IVT northwesterlies” in Fig. 15), which reduces the amount of moisture transported to the region. During cloud band events, the southerly anomalies over the west flank of the anomalous cyclonic circulation further reduce the IVT from the Amazon, resulting in lower precipitation rates during the events in the CPRCMs. Due to the spatial extent of the domain, it is possible that part of the circulation biases over subtropical SAm could be inherited from the forcing model since the flow does not have sufficient distance from lateral boundaries in order to be systematically modified by any local convection.

Even though not described here, part of the dry bias over SESA could be explained by the biases in basic state cloudiness and precipitation over northern SAm. The tropical North Atlantic Ocean is an important source of moisture to the Amazon (Builes-Jaramillo et al. 2022) and subtropical SAm (Yang and Dominguez 2019). The trade winds from the North Atlantic Ocean drive the cross-equatorial flow and the moisture transport into the Amazon (Drumond et al. 2014; Builes-Jaramillo et al. 2022). This flow is also strongly related to the onset of the South American Monsoon System over the tropics (Wang and Fu 2002). As the flow reaches the Andes, it turns counter-clockwise towards the subtropics, advecting moisture from the Amazon into the region, where it contributes to 16–30% of the summer precipitation (Yang and Dominguez 2019; Leyba et al. 2023). In addition to being a source of moisture, there are indications that the intensity of the cross-equatorial flow over the Amazon could modulate the intensity of the northwesterly Low-Level Jet, even though the link between the two systems needs to be further explored (Wang and Fu 2002; Builes-Jaramillo et al. 2022). Thus, biases in the lower-level winds over northern SAm can affect the amount of moisture reaching the Amazon (“IVT Noth Atlantic” in Fig. 15), the onset of the monsoon season, and the transport of moisture towards the subtropics. These circulation biases are present only in the convective-permitting simulations and are similar in the UKMO and NCAR WRF CPRCMs. Here, we suggest that it could be related to the extension of the CPRCM integration domain over the tropical North Atlantic, but this hypothesis should be further explored.

In conclusion, the results presented demonstrate that the first continental-scale convective-permitting regional climate model simulations over SAm reproduce the main characteristics of the rainy season, improving the representation of the cloud band seasonality and frequency despite overestimating (underestimating) the intensity of the precipitation in the tropics (subtropics). Improvements in simulating the seasonality of cloud bands further demonstrated the strength of event-based approaches to evaluate the simulations. This type of approach can be useful when studying the occurrence of extreme events, circumventing some of the shortcomings of CPRCMs, such as the precipitation intensity biases and the reduced length of the simulations. Given their high spatial resolution, these simulations can potentially improve the understanding of local and regional processes, such as the onset and demise of the rainy season or the occurrence of natural disasters related to excess or absence of precipitation. The accurate simulation of the cloud band events indicates that these simulations can be instrumental in understanding how future climate change will affect the occurrence of cloud band-related extreme events, as well as severe droughts related to the extended periods without events, as observed in the past (Coelho et al. 2016b, a). Simulations of future climate change scenarios are already available for the UKMO CPRCM and NCAR WRF CPRCM.

## Data Availability

The observational OLR data set data analysed during the current study is available at NOAA CDR (Lee and NOAA/CDR 2011). The CHIRPS precipitation data set is available at https://www.chc.ucsb.edu/data (Funk et al. 2015). ERA5 reanalysis data can be accessed through the Copernicus Climate Change Service (C3S) Climate Data Store (CDS; Hersbach et al. 2023). The Br-DWGD precipitation data can be obtained at https://sites.google.com/site/alexandrecandidoxavierufes/brazilian-daily-weather-gridded-data (Xavier et al. 2022). The Met Office Unifed Model (UM) CPRCM simulations are available from KH and RK on reasonable request (Halladay et al. 2023). Hourly output for the NCAR-WRF simulations for select variables is available at NCAR CISL Research Data Archive (Rasmussen et al. 2022).
